# Mineral Composition of Traditional Non-GMO Soybean Cultivars in relation to Nitrogen Fertilization

**DOI:** 10.1155/2020/9374564

**Published:** 2020-06-22

**Authors:** Bogdan Szostak, Aleksandra Głowacka, Renata Klebaniuk, Anna Kiełtyka-Dadasiewicz

**Affiliations:** ^1^Institute of Animal Nutrition and Bromatology, University of Life Sciences in Lublin, 13 Akademicka Street, 20-950 Lublin, Poland; ^2^Department of Plant Cultivation Technology and Commodity, University of Life Sciences in Lublin, 15 Akademicka Street, 20-950 Lublin, Poland

## Abstract

Soybean is widely used as food. Genetic factors, as well as agrotechnical procedures, affect the yield and quality of soybeans. The subject of our research was the synchronization between soil N supply (from both mineralization and fertilization) and crop N demand. The aim of the research was to determine the effect of the cultivar and nitrogen application on the seed yield and mineral content in soybeans. Two non-GMO soybean cultivars (Amandine and Merlin) and four mineral nitrogen fertilizers ((A) N 0, control; (B) N 30:0, 30 kg N ha^−1^ before sowing; (C) N 0:30, 30 kg N ha^−1^ at BBCH 73–75; (D) N 30:30, 30 kg N ha^−1^ before sowing and 30 kg N ha^−1^ at BBCH 73–75) were tested. The highest soybean yield was obtained following nitrogen application at a rate of 60 kg ha^−1^. The genetic factor was found to significantly influence the content of some macronutrients (P, K, and Mg) and micronutrients (Cu, Mn, and Fe). In general, the Merlin cultivar had better macronutrient parameters except nitrogen, while Amandine had a higher content of Cu and Fe. Nitrogen fertilization decreased the content of P, K, and Zn in the soybeans but significantly increased the content of Ca, Mg, Cu, and Mn.

## 1. Introduction

Due to its wide application in various industries, soybean (*Glycine max* (L.) Merrill) is grown in many regions of the world, mainly in North and South America and in Asia. The largest exporters of soybeans are the United States, Brazil, and Argentina. These countries together account for over 95% of global soybean exports and more than 75% of the global crop area of this plant [1]. In the 2016/17 season, imports of soybeans to EU countries amounted to 14.7 million tonnes. Poland imports over 2 million tonnes of soybean meal annually [2]. It is currently estimated that about 79% of the soy available on the market is derived from GM varieties [3]. The demand for conventional soybean varieties is the greatest in highly developed and prosperous European countries and Japan, accounting for about 10% of the total soy trade in the world.

The increased interest in soybeans in regions that do not have a long tradition of soy cultivation, including Poland, is due to the high price of soybean meal, changing climatic conditions, and increasing improvement in soybean yield. Attempts are also made to find ways to limit the use of genetically modified soybean meal. For this reason, recent years have seen increased interest among researchers and practitioners in improving varieties, adapting various soy varieties to different climate conditions, and establishing appropriate agrotechnical procedures for them [4–6]. It seems likely that the high genetic diversity of soybeans and many years of agrotechnical work are making it possible to increase the area of soybean cultivation in regions with less favourable conditions for this plant.

Soybeans are a valuable component in the production of animal feed, and soy products are becoming increasingly popular in the human diet as well. They are distinguished by a high content of proteins and fats, which constitute about 60% of the dry weight of the seeds. Their nutritional value is enriched by a large quantity of unsaturated fatty acids, B vitamins, and minerals, such as nitrogen, potassium, magnesium, iron, calcium, and phosphorus [1, 6]. The nutritional value of soybeans depends mainly on habitat factors and appropriate agrotechnical procedures, in which mineral fertilization plays an important role [7, 8]. Soy requires a large amount of nitrogen due to the high protein concentration in the seeds, about 35–40%. Biological binding of nitrogen only satisfies about 50–60% of soy demand for nitrogen, which is sufficient to obtain 80–90% of the soy yield possible with adequate nitrogen fertilization [9–11]. According to Salvagiotti et al. [12], nitrogen fertilization increases soy yield mainly in cases where soil and biological nitrogen fixation do not ensure sufficient uptake of this nutrient. Results obtained by La Menza et al. [13] suggest that N fixation alone is not sufficient to meet soybean N demand in production environments with high yield potential. N supply will likely become a major yield-limiting factor in soybean production systems with high yield potential as farmers continue to fine-tune their agronomic practices and adopt higher-yielding cultivars. In the case of nitrogen fertilization of soy, not only the amount but also the time of application is important. Zainab et al. [14], in a study on the response of soy to nitrogen fertilizer applied at various time periods at a level of 60 kg ha^−1^, obtained the highest seed yield when a quarter of the nitrogen fertilizer was applied before sowing and the rest at the start of the seed-filling stage. Bender et al. [15] also claimed that providing soy with available nutrients, including nitrogen, in the late generative development period made it possible to meet its requirements for reproduction to exploit the yield potential of new cultivars.

The effect of varied nitrogen fertilization on the efficiency of nitrogen fixation by rhizobia, yield, protein, and fat contents in seeds and their quality has been the subject of numerous studies [4, 7, 10, 12, 16, 17]. Few studies have dealt with the effect of nitrogen application on the mineral composition of soybeans. Kandil et al. [18] reported that 120 kg of nitrogen applied at the start of soy increased the content of nitrogen, phosphorus, manganese, and zinc in the seeds. The magnitude of these changes was influenced by the type of nitrogen fertilization, with ammonium nitrogen being found to be the most effective. Sohrabi et al. [19] have found that the application of nitrogen at 50 or 100 kg at the R4 stage (full pod) significantly affected only the content of nitrogen in soybeans in proportion to the amount used and manganese content in the case of the higher application rate. It had no significant effect on the content of the other elements tested, i.e., potassium, phosphorus, sodium, iron, zinc, or copper. The studies cited above were conducted in countries with a warm climate, i.e., Iran or Egypt. Experiments conducted in temperate climates and with newer varieties are very few. It may also be significant that most research has been conducted on glyphosate-resistant soybean varieties, which according to many authors may affect the uptake and content of elements in soy biomass and seeds [20–22]. Furthermore, new cultivars with high yield potential may differ in their uptake and accumulation of elements [15].

Soy is an important source not only of protein but also of fats, vitamins, isoflavones, and minerals [5, 7]. Plants require 17 nutrients that are considered essential, each of which performs different functions enabling the plant to grow and reproduce [23]. The composition and nutritional value of crop plants are also very important for human and animal health [24–26]. The nutrient content in plants depends on environmental and varietal factors and can also be modified by agrotechnical measures [27, 28]. Climate is one of the most important factors affecting agricultural production every year, even in high-performance and technologically advanced agricultural areas [29]. According to Kumagaia and Sameshima [30] and Żarski et al. [31], weather conditions in the growing period are crucial for the development of soybean, which is a thermophilic plant.

As mentioned above, there has been little research on the impact of nitrogen fertilization on the mineral composition of soybean seeds. There is a need for such research, because soy is an important source of not only protein in the human diet and animal nutrition but also essential minerals. In addition, interest in soybean cultivation in Poland, as well as in other countries of the region, has been increasing significantly in recent years. According to FAOSTAT [1] data, the area of soybean cultivation in Europe, which was 1.96 million ha in 2009, had reached 5.69 million ha in 2017. The area of soybean cultivation in Poland, which was 288 ha in 2004, had reached 9300 ha in 2017 and 12,000 ha by 2018. Therefore, the subject of our research was the synchronization between soil N supply (from both mineralization and fertilization) and crop N demand. The aim of the research was to determine the effect of the cultivar and the level and time of application of nitrogen fertilization on the seed yield and the content of macro- and micronutrients in soybeans cultivated in southeastern Poland condition.

## 2. Materials and Methods

### 2.1. Site Description and Experimental Design

A field experiment was carried out in 2015 and 2016 in Frankamionka (50°43′34″N 23°39′11″), in Zamość County, Poland. The experiment was located on mineral soil with the granulometric composition of silty clay. The basic soil properties are presented in Table 1. The experiment was carried out in the split-plot design with four replications. Two factors were analysed in the experiment: (i) soy cultivar: Amandine and Merlin; (ii) nitrogen application: (A) N 0, control (without nitrogen); (B) N 30:0, 30 kg N ha^−1^ before sowing; (C) N 0:30, 30 kg N ha^−1^ at BBCH 73–75; (D) N 30:30, 30 kg N ha^−1^ before sowing and 30 kg N ha^−1^ at BBCH 73–75. The nitrogen dose resulted from fertilizer recommendations for legumes in Poland. The time of nitrogen application was determined based on the results of other authors [17, 19, 33].

The soy cultivars selected for the study are traditional cultivars, not genetically modified. Merlin matures exceptionally early (maturity group: 000++), is resistant to lodging, and has the best yield potential among the earliest varieties. It was first registered in 1998, but it is still being developed in many countries and achieves the best yield results in official tests and in field production. The Amandine cultivar is a new, early maturing variety (maturity group: 000) with high yield potential and exceptionally high protein content. Due to its light stigma colouring and excellent flavour attributes, it is attractive as edible soybean.

In the first year and second year of the experiment, soy was sown on 30 April. The sowing rate was 140 kg ha^−1^, and the interrow width was 20 cm. The size of the plots was 12.5 m^2^ (2.5 × 5 m) for sowing and 9 m^2^ (2 × 4.5 m) for harvesting. The distance between plots was 1 m. In both years, the forecrop for soybean was winter wheat. The soybean seeds used in the experiment had been prepared for sowing using FIX FERTIG technology. In a special technological process, the seeds are coated with rhizobia together with a glue that also acts as a preservative and protects against sunlight [34]. In autumn of each year, phosphate fertilizer was applied at 50 kg P ha^−1^ and potassium at 90 kg K ha^−1^ (P: triple superphosphate, K: potassium salt 60% K_2_O). Nitrogen was applied according to the experimental design in the form of ammonium nitrate with 34% N. Classical soil cultivation was carried out as recommended for soybean. After harvesting of the forecrop, shallow ploughing (8–10 cm) was performed, followed by harrowing and winter ridge ploughing at medium depth (22–25 cm). Spring tillage was limited to early-spring harrowing and presowing cultivation with a tillage unit equipped with a packer roller, which promotes water infiltration. Sowing was carried out with a mechanical seed drill for cereals.

To protect the plantation from weeds, herbicides recommended for soybeans were applied immediately after sowing: Sencor Liquid 600 SC (a.i. metribuzin) at 0.5 l ha^−1^ and Dual Gold 960 EC (a.i. S-metolachlor) at 1.0 l ha^−1^. Soybean was harvested with a combine at full maturity (BBCH 99) in the second third of September.

### 2.2. Meteorological Conditions

The weather conditions during the research period are shown in Tables 2 and 3. Based on the meteorological data, the Selyaninov hydrothermal coefficient was calculated (Table 4), according to the following formula:

(1) $$\begin{array}{c} k=\frac{\left(p\times 10\right)}{\Sigma t}, \end{array}$$

where *p* is the sum of ten-day monthly precipitation (mm) and Ʃ*t* is the sum of average daily temperatures from a ten-day period/month (°C).

Designations for ranges of coefficient values were adopted according to the scale developed by Skowera et al. [35].

The amount of rainfall was similar in both growing seasons and was lower than the long-term average. Particularly abundant rainfall was recorded in May and August of 2015 and in September of 2016. In 2015, very low rainfall was recorded in June and in the first two-thirds of August (Table 3). The Selyaninov index indicates that the period from June to August 2015, except for the second third of July, was dry or very dry. In 2016, the last third of May, the first and last third of June, and the second third of August were extremely dry or very dry (Table 4). The sum of temperatures in the period from April to September in the growing seasons ranged from 3008°C to 3119°C, while the long-term average was 2850°C (Table 2).

### 2.3. Laboratory Analysis

After harvest, the seed yield was determined at a moisture content of 15%. Then 2 kg of seeds were collected from each plot. The seed samples were ground in a laboratory mill and stored in sealed jars for analysis. The content of macronutrients, i.e., nitrogen (by Kjeldahl method according to CLA/PSO/13), phosphorus (by spectrophotometry according to CLA/PLC/28), potassium, magnesium, calcium, and micronutrients, i.e., zinc, copper, iron, and manganese (by Atomic Absorption Spectrometry with excitation in the air-acetylene flame according to CLA/ASA/2), were determined in soy seed samples. The results were converted to dry weight. The analyses were carried out at the Central Laboratory of Agroecology of the University of Life Sciences in Lublin and at the laboratory of the Institute of Animal Nutrition and Bromatology.

### 2.4. Statistical Analysis

The results were statistically analysed by the analysis of variance using STATISTICA 13 PL software (Tulsa, USA). Three-way analysis of variance (ANOVA) was carried out to determine the effect of year, nitrogen fertilization, and cultivar on the variability of soybean yield and mineral composition. Prior to the analysis of variance, the Shapiro–Wilk test was used to determine whether the variables had a normal distribution [36]. The test showed that the results for the content of magnesium, copper, manganese, iron, and zinc in the soybeans did not have a normal distribution, so the data were log-transformed and the analysis of variance was carried out using the transformed data. The effect of year, nitrogen fertilization, cultivar, and their interactions were analysed using a split–split-plot design with the year being designed as whole plots, nitrogen fertilization as subplots, and cultivar as sub–subplots. The analysis of variance revealed nonsignificant effects of year × nitrogen × cultivar interaction for all the parameters measured. The paper presents only significant effects of the following interactions: year × nitrogen fertilization, year x cultivar, and cultivar × nitrogen fertilization. As the interaction was not significant for seed yield, the main effect was presented. Differences between averages were determined using Tukey's test, at *p* < 0.05. Pearson's correlation coefficients were calculated to determine the relationship between the yield and nutrient content in the soybean seeds.

## 3. Results and Discussion

### 3.1. Yield of Soybean Seeds

**Figure 1:**
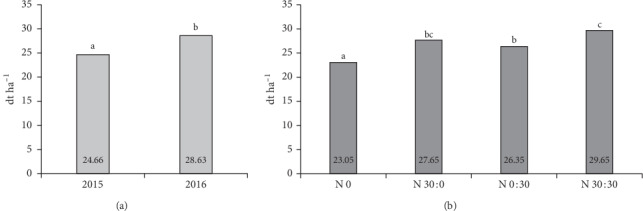
Effects of the year (a) and nitrogen application (b) on the yield of soybean seeds.

Crop yields are influenced by environmental, genetic, and agrotechnical factors. In our experiment, the highest yield of soybean seeds, 28.6 dt ha^−1^, was achieved in 2016 (Figure 1(a)), with uniform distribution of precipitation (Table 3). Soybean has high water requirements in the blooming and pod-filling stages [33]. In 2015, when the yield was lower (24.7 dt ha^−1^), all periods of June were extremely dry (Table 4), whereas there were major rainfall shortages in the first ten days of August, i.e., the seed-filling stage. There were no significant differences in seed yield between the tested cultivars. A lack of significant differences in the yield of different soybean varieties has been demonstrated by other authors [7, 37]. Nitrogen fertilization had a significant impact on the soybean yield. The lowest seed yield was obtained in the absence of nitrogen fertilization, and the highest for split nitrogen application at a rate of 60 kg ha^−1^: 30 kg ha^−1^ before sowing and 30 kg ha^−1^ at BBCH 73–75 (Figure 1(b)). Soybean has a relatively high N requirement, particularly during the seed-filling period, and biological N fixation may not supply sufficient N for the crop. Therefore, N application during the reproductive stages may improve yield [17]. Supplying N to the soybean plant during peak seed demand may supplement existing N resources, thus preventing premature senescence and increasing seed yield [19]. An increase in grain yield of soybean in response to N application has been observed in other experiments [4, 10, 12]. According to Gai et al. [38], the significant response of soybean grain yield to N supply at the time of sowing can be ascribed to a significant increase in root activity, the photosynthetic rate, and the leaf area index. The authors cited demonstrated that excessive or insufficient nitrogen application did not increase soybean grain yield, while the intermediate level of starter nitrogen fertilization (50 kg N) increased grain yield. Namvar and Sharifi [39] and Van Kessel and Hartley [40] have also suggested that the amount of starter N fertilizer of about 50 kg ha^−1^ is beneficial to legume plant development. Pampana et al. [41] also found that the response of legumes to nitrogen fertilization is dose-dependent. The authors cited reported that with N rates lower than 120 kg ha^−1^ reductions in nodulation and N_2_ fixation had no effect on above-ground growth and grain yield.

### 3.2. Macronutrients Content of Soybean Seeds

#### 3.2.1. Nitrogen

**Figure 2:**
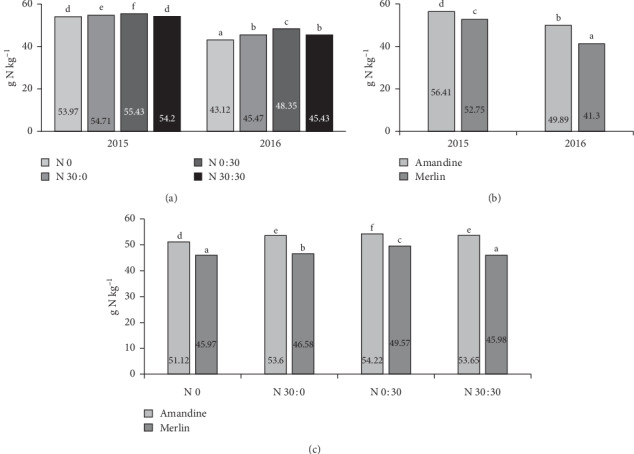
Effects of year × nitrogen application (a), year × cultivar (b), and cultivar × nitrogen application (c) on the nitrogen content in soybean seeds.

The analysis of the effect of the year of research and the cultivar shows that nitrogen content was higher in seeds of the Amandine cultivar in both 2015 and 2016 (Figure 2(b)). The Merlin variety accumulated significantly less of this element, and the differences were greater in 2016. The analysis of the interaction of years and fertilization reveals that, in all fertilization treatments, the soybeans contained more nitrogen in 2015. The highest nitrogen content in both years of the study was found in soybeans after the application of 30 kg ha^−1^ N at BBCH 73–75 (Figure 2(b)). Interestingly, in 2015, there were no significant differences between the treatments without nitrogen fertilization and with a split application of 60 kg ha^−1^. The analysis of the interaction between the cultivar and fertilization shows that both cultivars responded to the application of 30 kg ha^−1^ after emergence with increased nitrogen uptake relative to the control. The nitrogen content in the seeds of the Merlin cultivar from the control treatment and the treatment with 60 kg ha^−1^ of nitrogen did not differ significantly (Figure 2(c)).

Nitrogen is an important macronutrient for plants and is essential for protein synthesis. The increase in nitrogen content is an expected result in response to nitrogen fertilization [18]. Biological fixation only satisfies about 50–60% of soy demand for nitrogen [9–11]. As the N requirement of the soybean plant is not satisfied solely by fixation, a greater total amount of N must be supplied by the soil and/or remobilized from vegetative organs during grain filling [15, 42, 43]. In our experiment, nitrogen content in the soybean seeds also varied depending on the growing season. This was mainly due to variable meteorological conditions, as there was little variation in the properties of the soil on which the experiment was carried out in successive years (Table 1). The most important meteorological factors are temperature and water availability. According to Biel et al. [6], weather conditions substantially modify the nutrient content of soybeans and, thereby, reduce the impact of agrotechnical factors. Protein (and thus nitrogen as well) is promoted by higher average daily temperatures and light rainfall. In our experiment, the first growing period (2015) was dry, especially June and the first 20 days of September. According to Medic et al. [44], however, the relationship between water stress and soybean seed composition, i.e., nitrogen content, remains controversial. Some authors have reported an increase in protein content in the case of a soil moisture deficit [45, 46], while Spetch et al. [47] and Boydak et al. [48] observed a slight decrease in nitrogen content in water shortage conditions. An experiment conducted by Bellaloui and Mengistu [49] has suggested that the response of the soy plant to a soil moisture deficit may also depend on the cultivar.

#### 3.2.2. Phosphorus and Potassium

**Figure 3:**
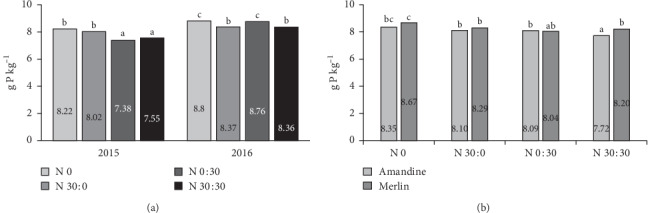
Effects of year × nitrogen application (a) and cultivar × nitrogen application (b) on the phosphorus content in soybean seeds.

The analysis of the effect of the year of research and fertilization on phosphorus content showed that the introduction of nitrogen at 30 kg ha^−1^ and 60 kg ha^−1^ (split application) significantly reduced the content of this element in the soybeans. The effect of a dose of 30 kg ha^−1^ depended on the time of application. Phosphorus content was significantly reduced by the application of 30 kg ha^−1^ N at BBCH 73–75 in 2015 and before sowing in 2016 (Figure 3(a)). For three of the four fertilization variants tested, the phosphorus content in soybeans was higher in the second year of the study. A significant difference in the reaction of the cultivars to nitrogen fertilization was observed only in the case of the application of 60 kg N ha^−1^ (Figure 3(b)). In this fertilization treatment, the Merlin cultivar had a higher content of phosphorus than Amandine. In general, the changes in phosphorus content in soybeans under the influence of the test factors were small, ranging from 7.38 to 8.86 g kg^−1^.

**Figure 4:**
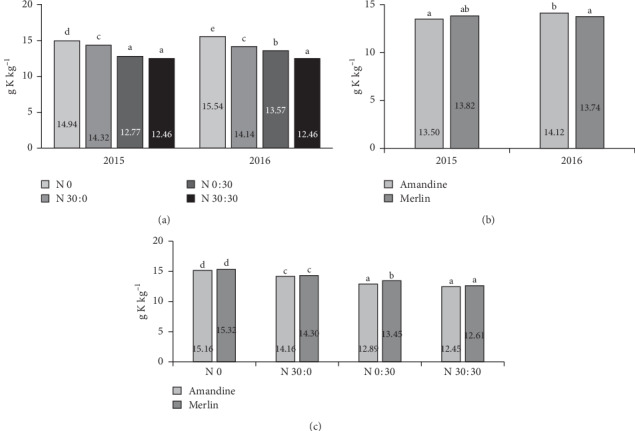
Effects of year × nitrogen application (a), year × cultivar (b), and cultivar × nitrogen application (c) on the potassium content in soybean seeds.

The content of potassium in the soybeans was influenced by the interaction of the experimental factors. In 2016, the Amandine cultivar contained more K than the Merlin variety, while in 2015 the cultivar had no significant impact (Figure 4(a)). In general, in both years of research, the use of nitrogen fertilization reduced the potassium content in the soybeans. In both the control treatment and the treatment with 30 kg ha^−1^ applied at BBCH 73–75, the content of potassium was significantly higher in 2016 than in 2015 (Figure 4(b)). A significant interaction of fertilization and cultivar was noted only in the treatment in which 30 kg ha^−1^ of nitrogen was applied as a top dressing (Figure 4(c)).

In a study by Kozak et al. [50], the highest content of phosphorus and potassium in soybeans was recorded in a growing season characterized by an exceptionally warm period from flowering to seed maturation. According to the authors, this may indicate that these nutrients may play a significant role in reducing moisture deficiencies in soy development. Tanguilig et al. [51] have also suggested that soybean plants maintain turgidity under drought by lowering the transpiration rate and maintaining uptake of nutrients, especially K, which is an important regulatory nutrient involved in osmotic adjustment. In the present study, in which the growing seasons had a similar temperature distribution, the content of phosphorus and potassium (for two fertilization variants) was significantly higher in 2016, in which only June was warmer. In addition, the Selyaninov index indicates that the period from June to August 2015, except for the second third of July, was dry or very dry. These results were in contrast to the findings of Samarah et al. [52], who reported a significant increase in P concentration in soybean seeds cultivated in drought conditions from the beginning seed (R5) to the full seed (R6) growth stage. The discrepancies in the results suggest that seed nutrient concentration could be highly responsive to the timing, duration, and severity of drought stress. On the other hand, phosphorus is relatively immobile in soils. Plants may have limited uptake of phosphorus if P is too far from the roots or if root growth is inhibited. Low soil moisture may decrease the availability of P and reduce its uptake [53].

Kozak et al. [50] stated that among the macronutrients determined in the soybeans only the content of phosphorus was significantly determined by the cultivar. However, in studies conducted by Biel et al. [54], the varietal factor did not affect the content of phosphorus in soybeans but significantly differentiated the content of potassium in the seeds. In Merlin cultivar, it was 20.48 g kg^−1^, whereas in Aldana, 22.63 g kg^−1^. In our study, potassium content in both tested cultivars was much lower. In general, the genetic factor did not significantly affect the content of phosphorus and potassium in the soybeans. According to Rigo et al. [26], potassium is the most stable nutrient for soybean cultivars.

In the study by Jarecki and Bobrecka-Jamro [7], the initial nitrogen application of 25 kg ha^−1^ did not significantly affect the content of phosphorus and potassium in soybeans. Other authors also found no significant effect of nitrogen fertilization on the content of phosphorus and potassium in soy seeds [19]. In our experiment, nitrogen application significantly reduced potassium content in the soy seeds. Nitrogen fertilization also had a significant impact on the soybean yield (Figure 1(b)). According to Cakmak [55], high-yielding crops may contain smaller amounts of nutrients than crops that produce less biomass, because they are “diluted” in the biomass of the plant. This phenomenon is supported by Pearson's correlation coefficients. The potassium content was negatively correlated with the seed yield (*r* = −0.454, *p*=0.05).

#### 3.2.3. Calcium and Magnesium

**Figure 5:**
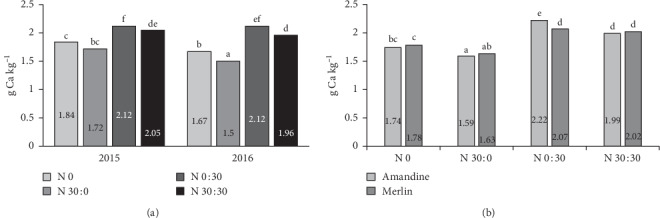
Effects of year × nitrogen application (a) and cultivar × nitrogen application (b) on the calcium content in soybean seeds.

According to Moraghan et al. [56], the calcium content in pulse plants is low. Furthermore, its availability is limited by oxalates and phytates. The content of calcium in soybeans can be influenced by the cultivar, fertilization, and weather conditions. In our experiment, the average content of calcium was 1.95 g kg^−1^ dw. This is much higher than the content reported by Biel et al. [54] for the Merlin cultivar (0.83 g kg^−1^) and similar to that found by Kozak et al. [50]. The analysis of variance confirmed the significant interaction of the year of research and nitrogen application in determining calcium content in soy. In both 2015 and 2016, the highest calcium content was observed after applying 30 kg ha^−1^ of nitrogen as a top dressing. Significant differences in the effect of nitrogen fertilization in individual years of research were found in the treatments in which 30 kg ha^−1^ of nitrogen was introduced before sowing (Figure 5(a)). Samarah et al. [52] found that drought stress treatments increased seed concentrations of Ca compared to the well-watered treatment and that the increase in mineral concentration under drought stress was not due to the reduction in dry matter accumulation. In our experiment, calcium content was higher in the first year of the experiment, when rainfall was very low in June and in the first two-thirds of August. However, this relationship was observed only for the control treatment and when 30 kg ha^−1^ N was applied before sowing. As in the case of potassium, a significant interaction of nitrogen fertilization and the genetic factor was observed only when 30 kg ha^−1^ N was applied at BBCH 73–75. The Amandine cultivar accumulated more calcium than the Merlin variety (Figure 5(b)).

**Figure 6:**
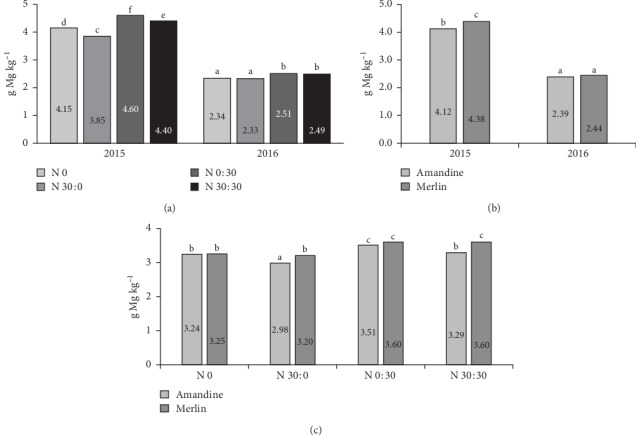
Effects of year × nitrogen application (a), year × cultivar (b), and cultivar × nitrogen application (c) on the magnesium content in soybean seeds.

In a study by Moraghan et al. [56], the magnesium content in the seeds of 12 soy varieties ranged from 1.67 to 2.23 g kg^−1^. Biel et al. [54] report an average magnesium content of 2.09 g kg^−1^ in soybeans of the Merlin cultivar. In our study, magnesium content in both tested cultivars was much higher, especially in the first year of the experiment (2015). In the case of magnesium, a significant interaction of the year of research and the genetic factor was observed (Figure 6(b)). In 2016, the magnesium content in the seeds of the two cultivars did not differ significantly, while in 2015 significantly more magnesium was found in the seeds of the Merlin variety.

Differences in the effect of the growing season or nitrogen fertilization on the content of individual macronutrients may be partly due to differences in the rate of uptake of these nutrients by soy plants. Bender et al. [15] found that almost three-quarters of total K uptake occurred prior to the onset of seed filling, while the uptake of P, Ca, and Mg was more evenly distributed over vegetative development and seed filling. Sale and Campbell [57] also report varied uptake and transport of nutrients to soybeans. Accumulation is rapid during the early seed-filling stage and slower during the late seed-filling period. According to the cited authors, at the onset of leaf ageing, an average of 80% of the final nutrient content is transferred to the growing seeds, but the percentage of the final content depends on the nutrient. At the onset of leaf ageing, the soybeans had accumulated nearly 90% of their iron but only 76% of their Ca. A research conducted by Bender et al. [15] suggests that two sources of nitrogen and phosphorus are used for soybean seed filling: soil absorption and remobilization of vegetative tissues. The authors demonstrated that over half of the total N accumulation and more than 45% of P accumulation occurred after the onset of seed filling. In addition, 65% and 32% of leaf nitrogen and stem nitrogen, respectively, measured near the beginning of seed filling, were remobilized to meet the N demand of filling seeds. A similar pattern occurred for P; approximately two-thirds of measured leaf and stem contents were remobilized to developing seed tissues. Pampana et al. [43] also stated that nitrogen remobilization was crucial in providing N to the seeds of chickpea, pea, and lupin (half of seed N content), but it was less important in the field bean (one-third). The authors suggested that improving grain legume yield requires either reduced N remobilization or enhanced N supply; thus a useful strategy is to select cultivars with high postanthesis N_2_ fixation or to add mineral N at flowering.

#### 3.2.4. Micronutrients

**Figure 7:**
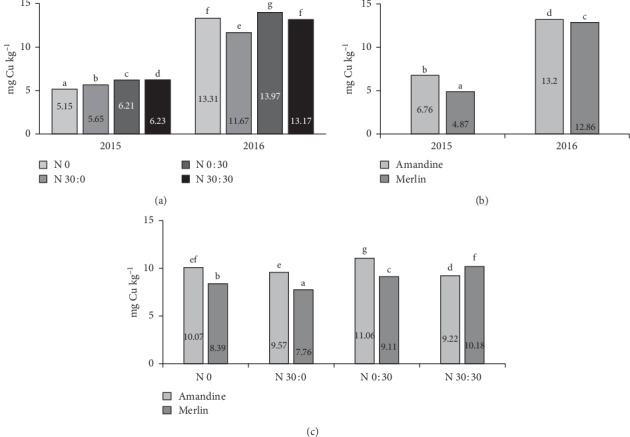
Effects of year × nitrogen application (a), year × cultivar (b), and cultivar × nitrogen application (c) on the copper content in soybean seeds.

Micronutrients such as iron, zinc, copper, and manganese perform various important functions in plants, animals, and humans. In general, the content of micronutrients in the soybeans tested in the experiment corresponded with values reported by other authors [26, 54, 58]. The uptake of micronutrients by plants depends on various factors, such as the species and even the cultivar, the stage of plant development, environmental conditions, and agrotechnical procedures [54, 59, 60]. In our study, a significant influence of the cultivar on Cu content in soybeans was noted in both years of research (Figure 7(a)). The Amandine cultivar had a higher content of this micronutrient in the seeds than the Merlin cultivar, but in 2015, these differences amounted to 39%, as compared to only 3% in 2016. The analysis of variance confirmed the significant influence of the interaction of the year of research and fertilization on the Cu content of soybeans (Figure 7(b)). In 2015, nitrogen application generally increased the content of this micronutrient in the seeds. In 2016, the effect of fertilization treatment was inconclusive. The application of 30 kg ha^−1^ before sowing reduced the amount of Cu in the seeds relative to the control treatment, while the same amount applied as top dressing increased the content of this micronutrient. The analysis of the effect of fertilization and cultivar reveals that the seeds of the Amandine cultivar had a higher content of Cu in all fertilizer treatments, with the exception of split application of 60 kg ha^−1^ (Figure 7(c)). These results are consistent with research by Biel et al. [54], who reported that Aldana soybeans accumulated more copper than seeds of the Merlin cultivar. The content of Cu in the seeds of the cultivars in 2015 ranged from 4.86 mg kg^−1^ to 6.48 mg kg^−1^and was similar to the values reported by Biel et al. [6]. In 2016, the Cu content in the seeds of the cultivars was much higher, ranging from 12.86 mg kg^−1^ to 13.20 mg kg^−1^, but did not exceed the values given for soy by other authors [59, 61]. Such substantial differences in copper content may have resulted from weather conditions in different growing seasons (Tables 2 and 3). According to Stanisławska-Glubiak and Korzeniowska [62], copper is arrested in soil by organic matter and released during its decomposition. Soil moisture is an important factor affecting the decomposition of organic matter and, consequently, the release of copper to the soil solution and the availability of this element to plants. However, in a study conducted by Samarah et al. [52], copper content in soybean seeds was increased by drought stress conditions.

**Figure 8:**
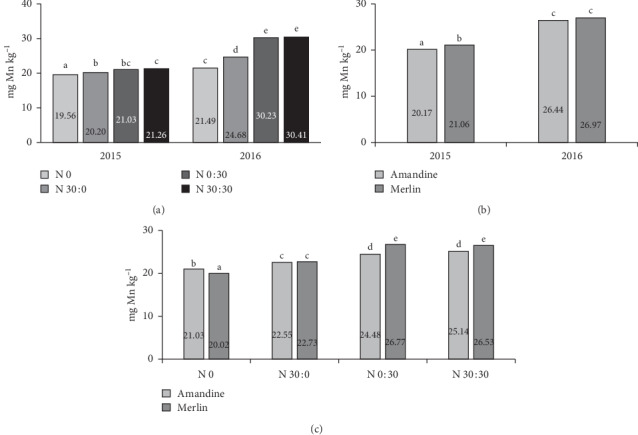
Effects of year × nitrogen application (a), year × cultivar (b), and cultivar × nitrogen application (c) on the manganese content in soybean seeds.

The content of Mn was significantly higher in the seeds of the Merlin variety, but only in 2015. In 2016, no significant impact of the genetic factor was found (Figure 8(a)). The analysis of the influence of the year of research and fertilization shows that in 2015 nitrogen application slightly but statistically significantly increased the content of Mn in the seeds relative to the control treatment (Figure 8(b)). However, there were no significant differences between the individual variants of nitrogen application. In 2016, the effect of fertilization was more pronounced, and the most manganese was accumulated in the seeds in the treatments with 30 kg ha^−1^ of nitrogen applied as a top dressing or split application of 60 kg ha^−1^. These results are consistent with research by Sohrabi et al. [19], who stated that, with the application of nitrogen fertilizer, Mn concentration in soybean seeds increased. Nitrogen fertilizer might promote Mn absorption by the roots and translocation of Mn from the root to the shoot of the plant, leading to increased Mn in the soybeans. The influence of cultivar and fertilization on Mn content is shown in Figure 8(c). In the control treatment, more of this microelement was found in the seeds of the Amandine variety. Following the application of 30 kg ha^−1^ before sowing, no significant differences were found between cultivars. In contrast, in the other two fertilizer treatments, the Merlin cultivar accumulated more Mn in the seeds.

**Figure 9:**
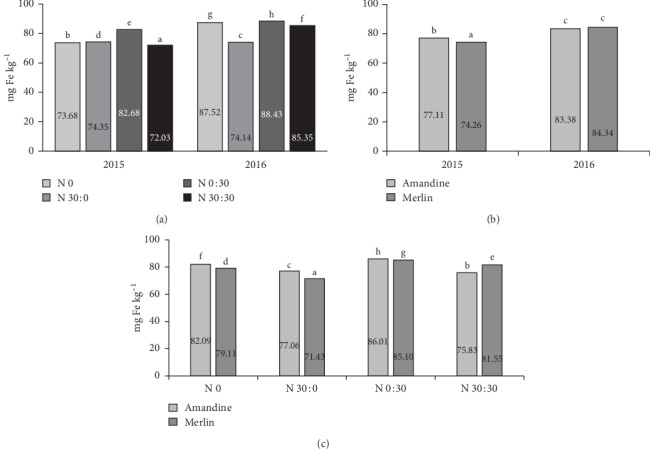
Effects of year × nitrogen application (a), year × cultivar (b), and cultivar × nitrogen application (c) on the iron content in soybean seeds.

According to Vasconcelos et al. [63], iron in soy plants plays an important role in the distribution and accumulation of other micronutrients. A significant effect of the genetic factor on Fe content in the seeds was only evident in 2015, when more of this microelement was found in the seeds of the Amandine cultivar (Figure 9(a)). The effect of the interaction of the years of research and fertilization on iron content was statistically significant (Figure 9(b)). In 2015, the application of 60 kg ha^−1^ N reduced the Fe content relative to the control treatment. In 2016, soybeans from the treatments with 30 kg applied before sowing and split application of 60 kg contained less Fe than soybeans from the control treatment. The application of 30 kg ha^−1^ N as top dressing increased the Fe content in the soybeans in each year of the experiment, but in 2015, the differences were greater. The analysis of the interaction of the cultivar and fertilization reveals that the Amandine cultivar had higher Fe content in the seeds than the Merlin cultivar (Figure 9(c)). Only in the treatment where 60 kg ha^−1^ N was applied was more of this microelement found in the seeds of the Merlin cultivar.

**Figure 10:**
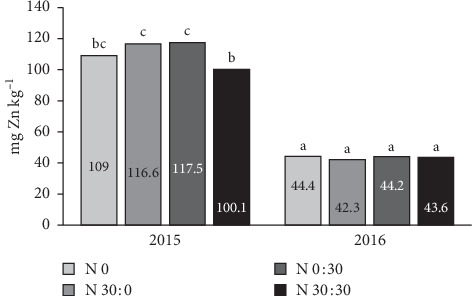
Effects of year × nitrogen on the zinc content in soybean seeds. Means marked with the same letters do not differ significantly.

The content of zinc in soybeans varied significantly depending on the interaction of the year of research and nitrogen fertilization (Figure 10). In 2016, Zn content was significantly lower than in 2015, and fertilization had no effect. In contrast, in 2015, significant differences were found only between the treatments with 60 kg ha^−1^ and 30 kg ha^−1^, but the time of application was not significant. The differences between growing seasons may be due to the greater soybean seed yield obtained in 2016 and the dilution effect [55]. Among the analysed micronutrients, only the content of zinc in the soybean seeds was negatively correlated with the seed yield (*r* = −0.622, *p*=0.05). The content of copper and manganese was positively correlated with soybean yield (*r* = 0.595 for Cu and *r* = 0.739 for Mn). There was no significant correlation between yield and iron content in the soybean seeds.

Micronutrient concentrations in different plant organs are associated with the stage of plant growth and the availability and mobility of the micronutrients. These can be transferred from the root, stem, leaf, and pod walls into developing seeds [58, 64]. The analysis performed in the present study revealed significant variation in the content of micronutrients in soy seeds. The differences can be attributed to climate, soil conditions, nitrogen fertilization, and plant variety. Differences in the content of individual micronutrients may be partly due to differences in the time and rate of nutrient uptake by soybean. Bender et al. [15] have reported that 75% of Fe uptake by soybean occurred before the onset of seed filling, whereas the uptake of micronutrients such Zn, Mn, and Cu was more evenly distributed during the vegetative and seed filling period. The authors cited demonstrated that more than one-half of leaf Cu was translocated to soybean grain tissues. Unlike the other micronutrients, copper is a nutrient with notable remobilization tendencies [65].

## 4. Conclusions

The results of this study confirm the impact of nitrogen fertilization on soybean yield. The lowest seed yield was obtained in the absence of nitrogen fertilization, and the highest for split nitrogen application at a rate of 60 kg ha^−1^.

The soybean cultivars responded differently to the level of nitrogen fertilization. Only the content of P and K in the seeds of both cultivars was the highest in the absence of nitrogen fertilization. Irrespective of nitrogen fertilization and the years of research, the Amandine variety had higher N content. The Merlin cultivar contained more P, Mg, Na, Cu, Mn, and Fe in the treatment with the application of 60 kg ha^−1^ of nitrogen. The Amandine cultivar accumulated more Cu and Fe than the Merlin variety in the control treatment and in the treatment with the application of 30 kg ha^−1^ of nitrogen, independently of the time of its application.

Irrespective of nitrogen treatment, the first growing season, with very low rainfall in June and in the first two-thirds of August, promoted the accumulation of N, Mg, and Zn in the soybeans. The content of P, Cu, Mn, and Fe was higher in the year with more evenly distributed rainfall.

The magnitude of the effect of nitrogen application on the content of macro- and micronutrients in soybeans was different in different years. In general, however, the application of nitrogen at 30 kg ha^−1^ at the seed-filling stage was most beneficial for the accumulation of valuable nutrients in seeds, i.e., N, Ca, Mg, Cu, Mn, and Fe.

## Figures and Tables

**Table 1:** Soil characteristics in the experimental field in a two-year study.

Soil characteristics	Year	
	2015	2016
Texture class^1^	Silty clay	Silty clay
Sand (2–0.05 mm)	19%	22%
Silt (0.05–0.002)	71%	69%
Clay (<0.002)	10%	9%
pH_KCl_	6.6	6.8
Total nitrogen (g kg^−1^)	1.3	1.4
Organic carbon (g kg^−1^)	19.7	18.9
Available forms (mg kg^−1^)		
Phosphorus	170	182
Potassium	197	205
Magnesium	51	57
Copper	45.2	40.6
Manganese	191.2	183.6
Iron	932	952
Zinc	9.0	10.2

^1^According to [32].

**Table 2:** Sum or average temperatures in months IV–IX as compared to the long-term means (1951–2010), according to the Meteorological Station in Felin, Poland.

Years	Decade	IV	V	VI	VII	VIII	IX	ΣV–IX
2015	I	4.5	13.5	18.6	21.4	25.1	15.4	1045
	II	8.6	13.2	17.7	19.8	22.0	17.6	1053
	III	13.2	13.1	16.8	20.7	20.6	12.9	1041
	Mean	8.7	13.3	17.7	20.6	22.6	13.3	2960
2016	I	10.7	13.6	17.0	18.9	18.9	19.2	1034
	II	10.6	12.2	18.2	18.7	16.8	16.3	986
	II	7.9	19.2	22.4	22.0	19.6	12.1	1100
	Mean	9.7	15.1	19.2	19.9	18.8	15.8	3009
Means for 1951–2010	I	5.7	11.5	15.5	17.1	18.1	14.2	868
	II	6.9	13.3	15.9	18.1	17.2	12.2	891
	III	9.3	13.6	16.7	18.0	15.7	10.8	898
	Mean	7.3	12.8	16.0	17.7	17.0	12.4	2543

**Table 3:** Rainfall in months IV–IX as compared to the long-term means (1951–2010), according to the Meteorological Station in Felin, Poland.

Years	Decade	IV	V	VI	VII	VIII	IX	ΣV–IX
2015	I	12.7	26.8	0.8	9.4	0	37.7	87.4
	II	2.8	8.9	8.1	23.5	0	32.2	75.4
	III	25.3	76.2	3.2	10.7	7.6	42.8	165.8
	Sum	40.8	111.9	12.1	43.6	7.6	112.7	328.7
2016	I	14.0	14.2	5.5	13.7	28.2	7.4	83.0
	II	13.6	21.6	35.5	49.8	5.9	0.0	126.4
	III	16.4	2.1	2.4	66.2	37.3	3.7	128.1
	Sum	44.0	37.9	43.4	129.7	71.4	11.1	337.5
Means for 1951–2010	I	13.5	16.5	21.8	23.6	25.6	20.3	121.3
	II	11.9	20.6	20.6	25.1	24.9	18.1	121.2
	III	13.0	22.7	22.5	32.1	19.2	14.5	124.0
	Sum	38.4	59.8	64.9	80.8	69.7	52.9	366.5

**Table 4:** Selyaninov's coefficient in months IV–IX compared to the long-term averages (1951–2010) according to the Meteorological Station in Felin, Poland.

Year	Decade	IV	V	VI	VII	VIII	IX
2015	I	2.8	1.8	0.0	0.4	0.0	2.2
	II	0.3	0.7	0.5	1.2	0.0	1.8
	III	1.9	2.7	0.2	0.5	0.4	3.3
	Mean	1.6	2.7	0.2	0.7	0.1	2.7
2016	I	1.3	0.9	0.3	0.7	1.4	0.4
	II	1.3	1.8	2.0	2.7	0.4	0.0
	III	2.1	0.1	0.1	3.0	1.9	0.3
	Mean	1.5	0.8	0.8	2.1	1.2	0.2
Means for 1951–2010	I	2.4	1.3	1.4	1.3	1.3	1.3
	II	1.7	1.5	1.3	1.4	1.4	1.5
	III	1.4	1.7	1.3	1.8	1.2	1.3
	Mean	1.8	1.5	1.4	1.5	1.3	1.4

## Data Availability

The data used to support the findings of this study are available from the corresponding author upon request.
